# The epidemiology and risk factors of respiratory syncytial virus and its impact on the timing of immunoprophylaxis

**DOI:** 10.7196/SARJ.2018.v24i1.202

**Published:** 2018-04-03

**Authors:** Prakash Mohan Jeena

**Affiliations:** 1 Department of Paediatrics and Child Health, Nelson R Mandela School of Medicine, University of KwaZulu-Natal, Durban, South Africa

## Editorial

Of an estimated 33.8 million episodes of respiratory syncytial virus (RSV) infections occurring in <5-year-olds globally each year, 3.4 million (10%) have severe disease that requires hospitalisation, of whom an estimated 60 000 - 199 000 die, 99% of these in developing countries.^[1,2]^ RSV is the most common cause of respiratory illness in infants worldwide, with an incidence of 30/1 000 child years, and has a mortality rate nine times that of influenza.^[1,2]^

The National Institute of Communicable Diseases of South Africa (SA) has been involved in several programmes related to surveillance of the syndromic recognition of respiratory illnesses (SRI).^[3]^ The pneumonia surveillance programme based at sentinel hospitals enrolled 3 746 patients, and tested 99% for RSV infection, 17% of whom tested positive. The RSV season (defined as >10% prevalence) started in week 7 - 8 of the year and continued through week 29, with the peak detection rate of 53% in week 18. The case fatality rate (CFR) for RSV was <1% (4/572). In another programme looking at influenza-like illness (ILI) based at two primary health clinics, in 2016, 1 668 patients with ILI were enrolled, and 1 645 (99%) samples were tested for respiratory pathogens. The overall detection rate of RSV was 6% (100/1 645), and detection rose above 10% in week 7 and was sustained at ≥10% until week 17.^[3]^ Similarly, data from a private-sector laboratory also confirmed the seasonality of RSV to be from late February to the end of June. Several factors have been implicated in the acquisition of RSV infection. These include geographic location (latitude and altitude) and climatic factors (temperature, barometric pressure, relative humidity, vapour tension, precipitation). Risk factors for serious RSV disease include prematurity (hospitalisation rate 25% - 30%, CFR 0.6% - 1.0%), congenital cyanotic heart disease (hospitalisation rate 59%, CFR 2% - 37%) and chronic lung disease (hospitalisation rate 60%, CFR 3.5% - 23%).^[4]^

In this issue of the journal, we have an informative article from the paediatrics department at Steve Biko Academic Hospital that describes viral infections identified from nasopharyngeal aspirates over a 4-year period.^[5]^ Of the 288 viral infections identified, RSV was seen in 162 isolates, diagnosed mainly through immunofluorescence (84%). The majority of RSV cases was seen in the <6-month-olds (63.4%), with a typical autumn-to-winter peak. Only 1.5% of the RSV cases who were tested for HIV were infected. There were eight deaths (4.9%) in the cohort, mainly in those aged >6 months, especially among those with the risk factors of prematurity or cardiac lesion. Although prematurity was identified as a risk factor for RSV, the exact gestational age where it poses the highest risk was not defined, although current international studies have shown the highest mortality rate in premature infants of 30 weeks’ gestational age.^[6]^ Thirty percent (*n*=49) of cases required admission to the intensive care unit (ICU), most of whom were <6 months of age, with an average length of ICU stay of 9.5 days. The survival rate of 95.1% is skewed compared with district and regional hospitals, as the unit serves as a tertiary referral centre. The high number of paediatric ICU (PICU) admissions for RSV cases <6 months is expected, as this is normally the age that has the highest numbers of PICU admissions. The linear increase in ICU numbers for RSV was not adjusted for confounding variables. Climatic risk factors for the acquisition of RSV could not be confirmed.

These new data on RSV in SA support previous studies showing a high burden but relatively low mortality of RSV infection in infancy. They confirm previous observations of low rates of coinfection in HIV-infected infants, with a seasonality that commences in late February and lasts to the middle of June. Risk factors for acquisition and severity include prematurity <30 weeks, chronic lung disease and congenital heart disease in the first year of life. Immunoprophylaxis of these groups at the appropriate time (between mid-February and mid-June) would be the most cost-effective response, providing the best value for money.
